# Numerical Simulation of Compressive Testing of Sandwich Structures with Novel Triply Periodic Minimal Surface Cores

**DOI:** 10.3390/ma18020260

**Published:** 2025-01-09

**Authors:** Alexandru Vasile, Dan Mihai Constantinescu, Andrei Ioan Indreș, Iulian Constantin Coropețchi, Ștefan Sorohan, Dragoş Alexandru Apostol

**Affiliations:** 1 Department of Strength of Materials, National University for Science and Technology POLITEHNICA Bucharest, Splaiul Independeţei 313, 060042 Bucharest, Romania; alexandru.vasile@mta.ro (A.V.); andrei.indres@mta.ro (A.I.I.); iulian.coropetchi@mta.ro (I.C.C.); stefan.sorohan@upb.ro (Ș.S.); dragos.apostol@upb.ro (D.A.A.); 2 Faculty of Aircraft and Military Vehicles, Military Technical Academy “Ferdinand I”, G. Coşbuc Blvd. 39-49, 050141 Bucharest, Romania; 3 Institute of Solid Mechanics of the Romanian Academy, Str. Constantin Mille No. 15, 010141 Bucharest, Romania

**Keywords:** TPMS, sandwich structure, finite element simulation, implicit modeling

## Abstract

Sandwich structures with triply periodic minimal surface (TPMS) cores have garnered research attention due to their potential to address challenges in lightweight solutions, high-strength designs, and energy absorption capabilities. This study focuses on performing finite element analyses (FEAs) on eight novel TPMS cores and one stochastic topology. It presents a method of analysis obtained through implicit modeling in *Ansys* simulations and examines whether the results obtained differ from a conventional method that uses a non-uniform rational B-spline (NURBS) approach. The study further presents a sensitivity analysis and a qualitative analysis of the meshes and four material models are evaluated to find the best candidate for polymeric parts created by additive manufacturing (AM) using a stereolithography (SLA) method. The FEA results from static and explicit simulations are compared with experimental data and while discrepancies are identified in some of the specimens, the failure mechanism of the proposed topologies can generally be estimated without the need for an empirical investigation. Results suggest that implicit modeling, while more computationally expensive, is as accurate as traditional methods. Additionally, insights into numerical simulations and optimal input parameters are provided to effectively validate structural designs for sandwich-type engineering applications.

## 1. Introduction

Sandwich structures that incorporate TPMS cores are a promising type of lightweight and robust material in the field of mechanical engineering. Characterized by their periodic geometry and minimal surface area, they offer a combination of high strength-to-weight ratio, excellent energy absorption, and adaptability to various design requirements. These structures have attracted significant interest for applications in aerospace, biomedical engineering, and structural mechanics due to their ability to efficiently manage mechanical loads while minimizing material usage. A comprehensive classification of the various types of sandwich structures featuring metamaterial cores is provided in references [1,2,3].

TPMS topologies, such as the Schwarz primitive, Schoen gyroid, and diamond surfaces, are defined mathematically through equations, enabling precise control over their geometrical parameters and relative density [4,5]. Their mathematical foundation makes them particularly suitable for optimization and integration into sandwich panels, where they act as cores between rigid face sheets. Recent advancements in additive manufacturing have facilitated the fabrication of these complex geometries, enabling fabrication of designs that were previously impractical [6,7,8].

Experimental studies alone, while providing valuable empirical insights, may not fully capture the complexities of the physical phenomena. Thus, a finite element analysis (FEA) serves as a complementary and indispensable tool for expanding the understanding derived from experiments. In addition, a correspondence between experimental data and FEA increases the confidence in the validity of the obtained results. This is especially important when complex topologies fabricated through additive manufacturing are considered, given the defects such methods could induce, altering their mechanical response [9].

Predicting deformation patterns, stress distribution, and failure mechanisms of TPMS structures under various loading conditions have received extensive interest. Recent relevant studies focus on FEA of the most common types of TPMS structures under compressive [10,11] and impact loading [12,13]. The effects of core geometry, material properties, and relative density on the overall performance of the structures was investigated for parts fabricated through AM of metallic [14,15] or polymeric materials [16,17]. Alternative optimization approaches, such as graded structures [18,19], or hybrid topologies [20,21], have also been proposed. The most commonly used types of FEA software used for simulating TPMS geometries are the *Abaqus* Explicit Solver [22,23,24,25] and *Ansys* Explicit Dynamics [26,27,28,29].

Implicit modeling allows for the quick generation of different topologies, facilitating the validation of FEA models against experimental data. Consequently, all other design variables are utilized to predict specific mechanical responses, as illustrated in the example described in [30]. To reduce the computational effort of simulating small and complex-shaped TPMS samples, studies tend to analyze only the behavior of a representative volume element (RVE) [26,31]. However, due to the lack of double symmetry and because the general failure mode is influenced by adjacent cells, an analysis of a larger domain is required and often performed [32,33].

This research article focuses on the numerical simulation and analysis of sandwich structures with novel TPMS cores, underlining the benefits of using implicit modeling. A total of eight novel TPMS structures and one stochastic geometry are verified while comparing results with experimental data. The design and fabrication of the topologies were described in more detail in [34]. By presenting computational tools and determining how different input parameters and material models influence FEA results, this study seeks to contribute to the development of lightweight, efficient, and customizable structural solutions for advanced engineering applications.

## 2. Materials and Methods

Due to the complex shape of the proposed shell-based TPMS samples, the walls of the cells come into contact with each other at high strain values. Thus, an analysis over the entire 10 mm deformation range (33% strain) which was considered experimentally [34] was impossible to perform in the *Static Structural* module of *Ansys*. So, separate analyses were carried out. The first one captured only the sample response in the elastic region and the onset of yielding and was performed in the *Static Structural* module, neglecting any self-contacts and gradual stiffening. The study was continued in the *Explicit Dynamics* module where the samples were simulated up to the deformation considered in the experimental tests, i.e., 10 mm.

### 2.1. CAD Model for Simulation

Preparing the geometry for FEA is a critical endeavor for an accurate analysis. The initial step involves obtaining a comprehensive representation of the structure, through methods such as computer-aided design, implicit modeling, or three-dimensional scanning. Simulation accuracy depends on the fidelity of the geometry, which ensures reliable results for the investigated physical phenomenon [35].

Computer-aided design (CAD) and implicit modeling are two distinct approaches in geometric representation. CAD employs explicit parametric models with precise geometries defined by vertices, edges, and faces. In contrast, implicit modeling represents surfaces as scalar fields using mathematical functions. This approach is not as widely used in engineering but is more flexible for handling complex shapes and topological changes. Although it has advantages in terms of easy parametrization and visualization of topologies, its usage in FEA models can prove problematic [36]. Different methods have been proposed to reduce the number of triangles used in converting implicit models into CAD-neutral formats [31,37]. In this study, the usage of geometries obtained through both conventional CAD and implicit modeling is presented and compared.

**Figure 1:**
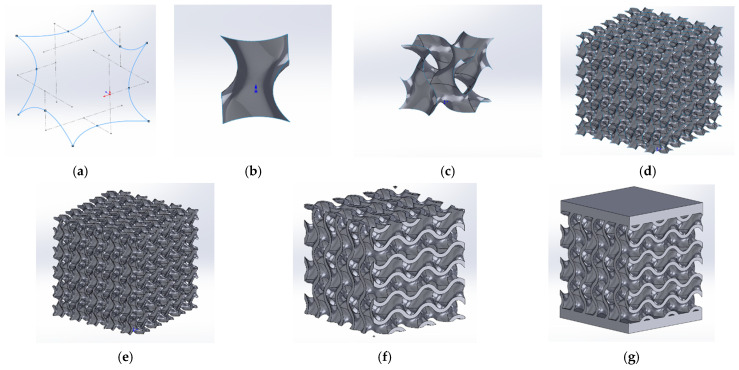
Construction of the CAD-type geometry of sample S1 (gyroid): (**a**) Surface contour; (**b**) Reference surface for 1/8 of RVE; (**c**) The surface of an RVE; (**d**) Multiplication for sample dimensions; (**e**) Obtaining a solid by thickening the walls; (**f**) Cutting to desired dimensions; (**g**) Obtaining the sandwich-type geometry.

Traditionally, in FEA, CAD-type geometries that are easy to integrate into numerical calculation models are used. This is possible, however, only when the samples are simple enough to be modeled either through parametrization based on mathematical functions or by a geometric approach. The stages of designing a CAD gyroid structure can be seen in Figure 1 and consist of the following: defining the reference tridimensional contour (a) and surface (b), making the necessary translations and multiplying to create the RVE (c), multiplying the RVE according to the proposed dimensions (d), applying the correct thickness of the walls (e), cutting the abnormal faces generated on the sides (f), and adding the face sheets of the sandwich structure (g).

The multitude and complexity of these operations help to underline the benefits of using implicit modeling, as all these steps can be replaced by changing the mathematical parameters of the model.

**Figure 2:**
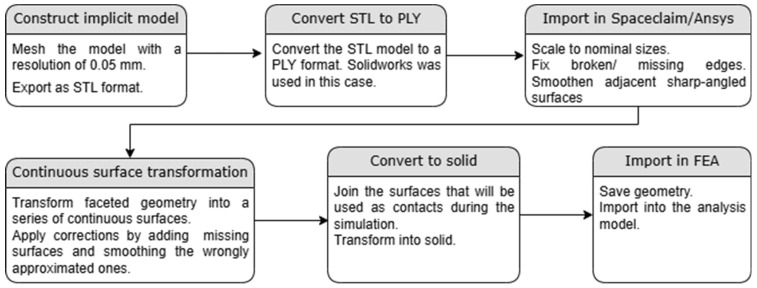
Procedure for obtaining solids usable in finite model analyses starting from implicit modeling.

In the case of complex geometries such as topologies S2–S9 defined in [34], modeling by using a CAD approach becomes problematic. When the functions used are complicated, the transition from the explicit form of the defining function f(x,y,z)=c to the parametric equations $x=f\left(t\right),\;y=g\left(t\right),\;z=h(t)$, used by conventional design software, is difficult. In Figure 2, the procedure that resulted from implicit modeling used in this paper to generate a complex model integrated into numerical analyses is proposed.

This was achieved through the following steps. First, the implicit topology was created in the *nTopology* (version 4.5.3) software. The implicit volume was meshed with a size of 0.05 mm, and the resulting geometry was exported in the STL format. This dimension was adopted because it is the same as the layer height that was used in the AM process. Then, the STL format is converted into a PLY format. We used *Solidworks 2023* software, as our observations indicated that this model produces a faceted body with fewer errors, requiring simpler further processing. The resulting format is then imported into the *Spaceclaim* module of *Ansys Workbench 2024 R1*. Because the PLY format does not retain information related to the dimensions of the sample, it is necessary to scale the imported part. This was initially performed for the gyroid geometry, for which the CAD format is also available. Samples being of the same size, the conversion factor remains the same for each subsequent sample. A faceted-surface-type geometry is thus obtained, consisting of surfaces specific to the mesh previously created. The next step is the correction of the discontinuous or missing edges and the smoothing of adjacent surfaces with sharp angles between them. This is necessary because the continuity errors appearing on the surface of the samples make the successful creation of a solid impossible. The geometry is transformed into higher-dimensional surfaces using the *Autoskin* feature. This transformation can generate approximation errors, leading to missing surfaces or erroneous edges. It was therefore necessary to fix the geometry by simplifying the sides of the surfaces, smoothing the wrongly discretized sides, removing the extra surfaces, joining the small surfaces, and filling the gaps. One step that simplifies the subsequent application of the boundary conditions is to join the surfaces to be used as contacts in the FEA, such as the top and bottom sheets of the sandwich structure. After this step, the conversion to a solid is performed, which can be further imported as geometry into the numerical analysis model.

### 2.2. Material Definition

The second challenge consists in defining, as accurately as possible, the behavior of the material used to manufacture the samples. Although the library of the *Mechanical* module of *Ansys* contains a large number of predefined material models, it does not address the majority of materials used in AM processes, especially SLA. Thus, to ensure the accuracy of the model, the mechanical properties of the commercially available photosensitive resin used (Tough 1500 v1—Formlabs—Somerville, MA, USA) were specified based on experimental data obtained from uniaxial tensile tests.

**Figure 3:**
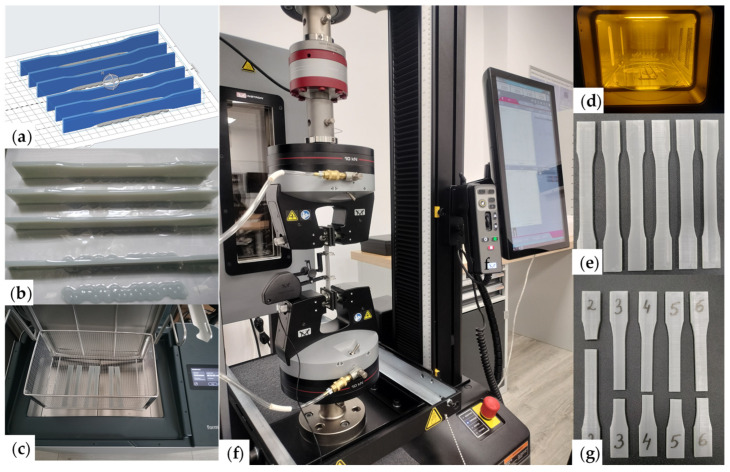
Fabrication of tensile samples and their uniaxial tensile tests: (**a**) Layout of the samples in the printing software; (**b**) Samples on the printing table; (**c**) Post-processing by washing with isopropyl alcohol; (**d**) Post-processing by treatment with ultraviolet radiation; (**e**) Printed samples; (**f**) The uniaxial testing configuration of the specimens; (**g**) Specimens after tensile tests.

Ten samples were fabricated using the same printing parameters and evaluated under uniaxial loading. Tensile tests were performed, according to the ASTM D638 standard [38], on an Instron 68TM-50 (Instron—Norwood, MA, USA) static and dynamic testing system with a 10 kN force cell and an Instron 2630-100 (Instron—Norwood, MA, USA) digital extensometer. Figure 3 briefly portrays the fabrication and testing setup of the tensile specimens. The conventional stress–strain curves were subsequently converted to true stress–strain curves.

**Figure 4:**
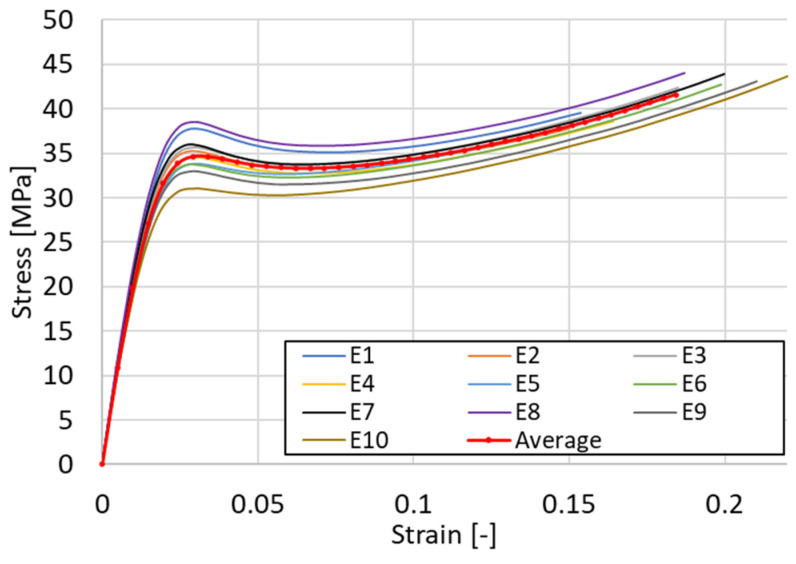
True stress–strain plots obtained through tensile testing of 10 specimens and the mean curve.

The red curve represented in Figure 4 shows the average (mean) true stress–strain curve for the 10 uniaxial tensile tests carried out. An initial region of linear elastic behavior of the material is observed up to a strain of 2%. After yielding, a strain-softening tendency is identified, where the slope of the curve becomes negative, and a necking zone can be observed on the specimen. The third region corresponds to a long hardening region, specific to polymeric materials, with an almost linear evolution until the final rupture of the specimen.

To use these curves as a reference behavior for numerical simulations, a separation between the linear elastic and non-linear behavior of the material is needed. Considering, based on the analysis performed on the homogeneity of the samples [39], that the material has isotropic properties, the first region was defined considering the mean value for the longitudinal (Young’s) modulus of elasticity of *E* = 1296 MPa and a Poisson’s ratio *ν* = 0.35, based also on the observations from relevant literature [40,41]. Next, using four material models presented below, the non-linearity of the material was approximated.

**Figure 5:**
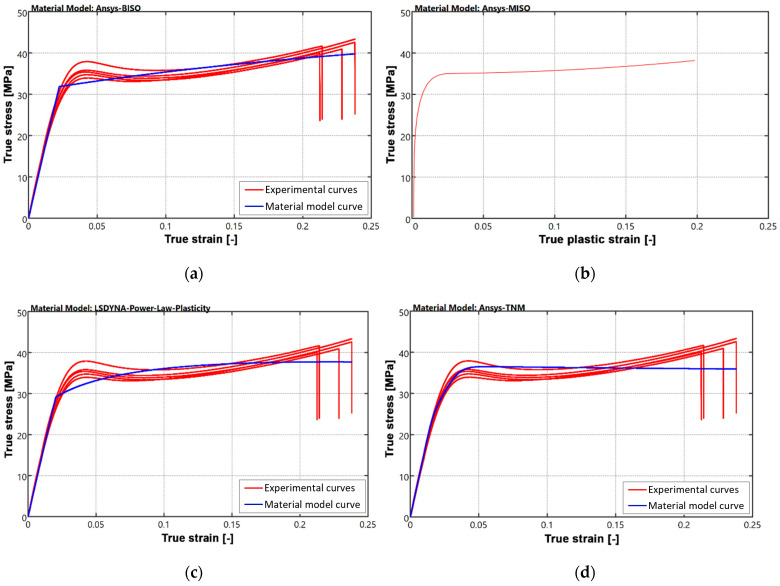
Material models proposed: (**a**) bilinear isotropic hardening; (**b**) multilinear isotropic hardening; (**c**) power law plasticity; (**d**) three-network model.

The simplest approach is to use a bilinear approximation of the curve, where the initial linear segment given by the slope *E* characterizes the elastic region, while the subsequent linear segment accounts for the hardening behavior after yielding and is given by the tangent modulus *E*_t_. The relatively constant hardening rate after yielding makes the model suitable for the studied polymeric material. The bilinear isotropic hardening model that approximates the experimentally obtained data is presented in Figure 5a, with the defining parameters given in Table 1.

A model that overlays the experimental curve with better accuracy is the *multilinear hardening plasticity*, which does not assume a constant slope and allows a more realistic representation of the material response by incorporating several linear segments. Its definition was achieved by introducing several points corresponding to the true stress–true plastic strain curve. Each segment corresponds to a distinct hardening stage, allowing the model to capture the evolving behavior of the material as it undergoes successive plastic deformations. Since the model does not allow for defining a negative slope, which is visible in the red curve presented in Figure 4, it was necessary to convert the true stress–strain evolution into a curve that is increasing continuously. The construction of a polynomial function that superimposes as much as possible over the real variation, described by relation (3) in Table 1, was achieved and is represented in Figure 5b. The other formulas used in defining the model of material are relations (1) and (2), where: σ_real_—true stress value, ε_pl_—plastic strain, ε_el_—elastic strain, *E*—Young’s modulus.

Considering the numerous material models available in FEA libraries used for numerical simulations and the complexity involved in defining them, it is beneficial to adopt simplifying approaches when working with more complex materials. Such a model is the *power law non-linear isotropic hardening* model, used in the case of materials that exhibit a non-linear hardening in the plasticity zone, defined by (4), where: *σ*_y_—yield stress value, *G*—transversal modulus of elasticity, and *N* = 0.15 constant exponent as defined in [42]. In Figure 5c, the resulting model curve is plotted together with the experimental data.

Working with any material model requires determining appropriate material parameters from experimental data. A good option is the use of the package *PolyUMod*^®^ (*Polymer FEM*, bought by *Ansys*) whose family of material models has incorporated advanced user-material models for finite element modeling of polymers, biomaterials, and other non-linear materials. To facilitate parameter extraction, *Polymer FEM* has developed *MCalibration*, which enables the semi-automatic extraction of pertinent material parameters from experimental data. This allows for the use of an iterative approach, in which the parameters and coefficients from the predefined material model formulas are modified until its curve approximates as accurately as possible the true strain–stress curves obtained experimentally by testing the tensile specimens. These parameters were then entered into the *Ansys* software *Workbench 2024 R1*, into the appropriate models, to be used in the FEA.

Due to the observations made after compressive testing, when the samples returned almost to their original shape within a couple of minutes, a material model that takes into account a viscoelastic behavior was also considered. The *three-network model (TNM)* states that three time-dependent viscous networks in molecular equilibrium give the mechanical response of the material, acting in parallel. In Table 1, the parameters that define the model, customized within *PolyUMod*, are manually adjusted and presented following the parameter notations found in *Ansys*. Figure 5d highlights the resulting curve. Volghin and Shishkovsky provide similar examples of the use of the model in [43] and Kumar in [44], for simulating the response of a part made from polylactic acid. The advantage of the model is that it allows capturing specific softening curves with negative slope that usually occur in polymers after yielding (Figure 5d).

### 2.3. Geometry Meshing

Another element that fundamentally influences the accuracy and reliability of FEA is the realization of a mesh adapted to the geometry and loading conditions, which ensures the faithful representation of the structural response [45]. The fineness, element type, and method used for meshing the model directly influence the convergence of the solution and the ability to capture the failure mode of structures with intricate topologies.

In the case of the proposed TPMS samples, although the CAD-type geometry could be meshed with quadratic elements, the models designed through implicit modeling presented errors or significantly reduced values for the mesh quality when trying to use the same order of elements. For this reason, to maintain consistent results, it was decided to discretize all the models using tetrahedron elements. The solid parts were discretized using Solid187 elements, while the contact area was constructed using Targe170 elements, which are specific to rigid materials and therefore applicable to the platens of the testing machine and Conta174 elements, which are specific for deformable elements and therefore applicable for the geometry of the metamaterial [46,47].

Creating a matching mesh between the elements of both surfaces in contact is crucial for accurately capturing their mechanical behavior. If this is easily obtained for the upper and lower surfaces of the specimen given by the contact between the platens and the face sheets of the sandwich structures, achieving compatibility between the elements of the metamaterial walls that come into contact with each other is difficult to realize. This is one of the reasons why convergence is often not reached at greater strain values.

Several meshing methods were verified to see which one works better for the proposed geometries. The simplest and most effective is the “*Automatic Method*”. However, it generated meshing errors in certain regions, consisting of several surfaces with large radii of curvature. The complicated nature of the topologies made the “*Multizone*” method exhibit serious implementation difficulties and unrepeatability between samples when separate meshing of the face sheets and the metamaterial core was attempted. The “*Patch Conforming*” method is well-suited for complex geometries, providing enhanced control over mesh quality. It enabled the successful meshing of all analyzed samples.

**Figure 6:**
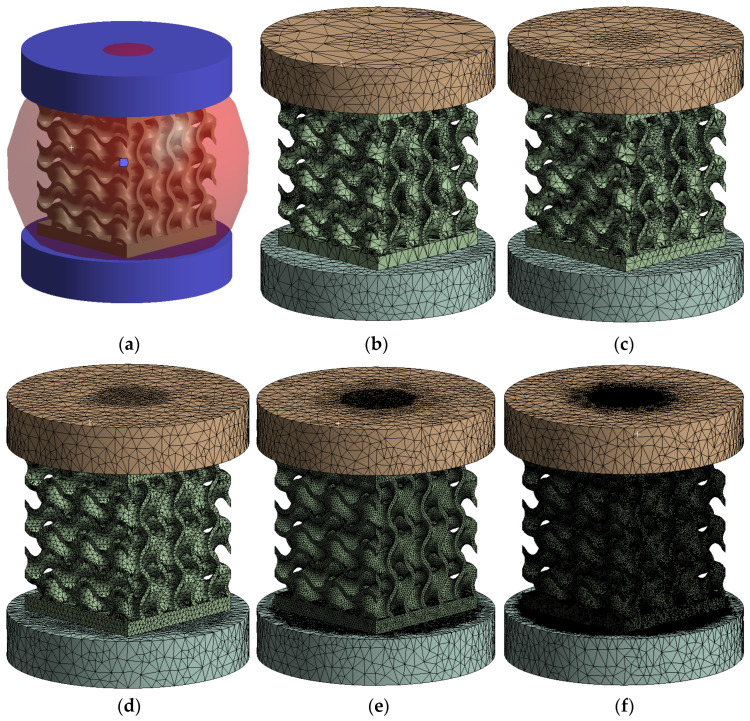
(**a**) Geometry of the gyroid model; (**b**) 3 mm maximum mesh element size; (**c**) 2 mm maximum mesh element size; (**d**) 1 mm maximum mesh element size; (**e**) 0.5 mm maximum mesh element size; (**f**) 0.3 mm maximum mesh element size.

A convergence study was carried out separately for the geometry of the test model assembly. The metamaterial solid was meshed with elements with a maximum size of 0.3 mm, 0.5 mm, 1 mm, 2 mm, or 3 mm, while the compression machine platens were discretized into two regions using spheres of influence, visible in Figure 6a. The minimum dimension of 0.3 mm was selected to have at least 3 elements along the thickness of the sample walls. The platen region in contact with the sample and its immediate vicinity was meshed with elements whose size coincided with that of the tested part. In the outer area of the platens the size was increased up to 10 mm.

To indicate the mesh quality, the “*Element quality*” index available in *Ansys* was used, where values close to 1 show elements formed from equilateral tetrahedrons, while values close to 0 indicate deformed elements with sharp angles. The latter introduces large errors in capturing the physical phenomenon. Figure 6b–f show the different wall thickness meshes considered for the sensitivity analysis.

### 2.4. Boundary Conditions

For the first static analysis, the *Static Structural* module of *Ansys* that aimed to capture the mechanical response in the elastic deformation zone until the onset of yielding is used. The following boundary conditions were considered: the contact between the platens of the testing machine and the faces of the sandwich structure was frictionless, the lower platen had all its degrees of freedom blocked, and a 2.5 mm vertical displacement was applied to the upper platen.

The second numerical simulation was carried out in the *Explicit Dynamics* module, where it was possible to take into account the self-contact that occurred between the walls of the samples over the entire 10 mm deformation range. In this case, to simplify the model, a 10 mm deformation was introduced to the upper surface of the TPMS structures, without considering in the model the platens of the testing machine. The bottom surface was considered fixed.

## 3. Results

Next, the results obtained from numerical simulations are presented. As previously mentioned, the geometries obtained through implicit modeling require an increased computational effort and a more complex meshing procedure. In order to gain a detailed understanding of the phenomenon and establish how the parameters used in the FEA model influence the results, several tests were carried out on the gyroid geometry, considered as a benchmark topology.

### 3.1. Influence of Mesh Size

**Figure 7:**
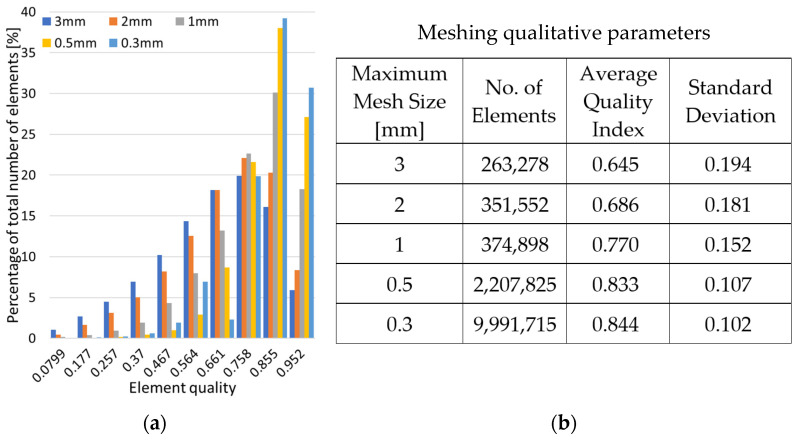
(**a**) Meshing quality index distribution; (**b**) Meshing qualitative parameters.

Initially, a convergence study was performed in order to analyze how the maximum size of the elements influence the final results. Figure 7a displays the percentage distribution of the number of elements of each mesh. Figure 7b shows the meshing qualitative parameters. It is observed that, contrary to expectations, there is no proportionality between the maximum size of the mesh and the number of elements into which the solid is discretized. This is due to the large elements generated in areas with large radii of curvature, which require a finer mesh in adjacent areas where the radius of curvature of the surfaces decreases. However, a positive trend in the average quality index and a negative trend in the standard deviation are observed as the refinement of the mesh increases. Also, the number of elements along the thickness of the geometry walls is important, noting that in the case of a mesh with an element size greater than 1 mm, a single element was generated throughout the thickness. This resulted in element shapes that significantly deviate from the ideal equilateral triangle shape typical of regular tetrahedron elements. As the mesh size is decreased, the walls are divided into more layers, which facilitates the creation of higher-quality elements along the surfaces.

An analysis of this type is useful because it became obvious that when the mesh refinement increased, the percentage of elements of higher qualities also increased significantly. Thus, the total number of distorted elements has been considerably reduced, leading to a lower probability of convergence issues of significant errors for the proposed gyroid model.

**Figure 8:**
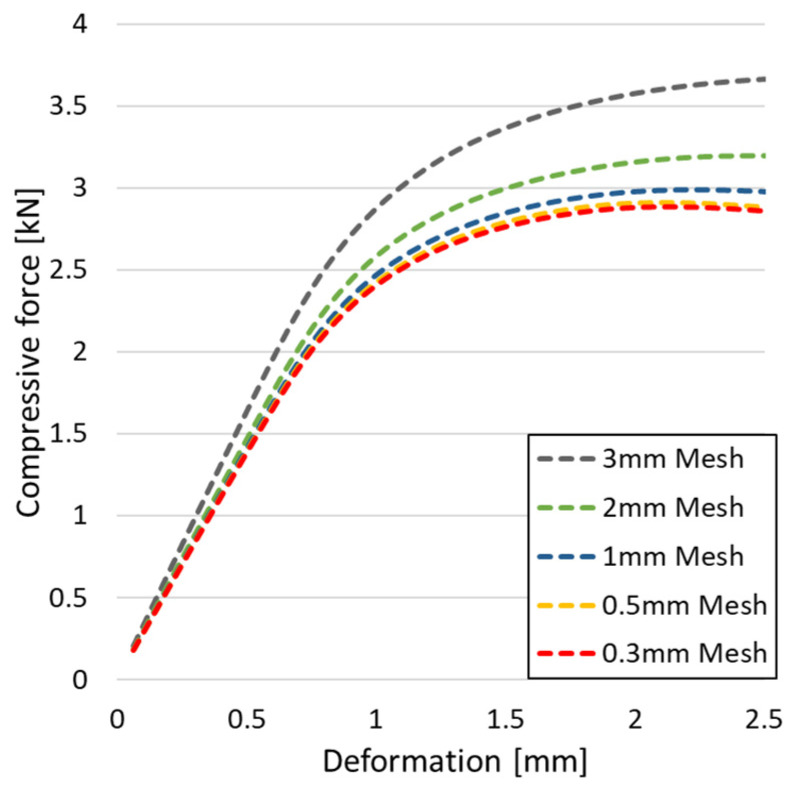
Evolution of the compressive force as a function of deformation for different mesh sizes.

Figure 8 shows the compressive force curves as a function of deformation for different mesh sizes. The study was carried out in the *Static Structural* module of *Ansys Mechanical*, on the gyroid geometry, not taking into account the self-contacts that occur during deformation. The material selected was the *Multilinear* model presented in Figure 5.

As the mesh refinement is increased, the value of the compressive force decreases significantly. If the model is composed of more elements, the value of the loading force is calculated more precisely. This is based on the increased ability to capture stress gradients more accurately across the part. There is, however, a point of diminishing improvement, where only computational time was added without a significant increase in model accuracy. For this reason, a mesh with a maximum size of 0.3 mm was adopted in order to provide a reasonable balance between convergence time and result accuracy. This mesh size was kept constant in all subsequent analyses.

### 3.2. Influence of Material Models

Due to the typical behavior of the polymer material, given by a higher yield stress followed by a softening region and stiffening at significant deformations, several material models were proposed to establish which characterizes the material behavior with the best accuracy.

**Figure 9:**
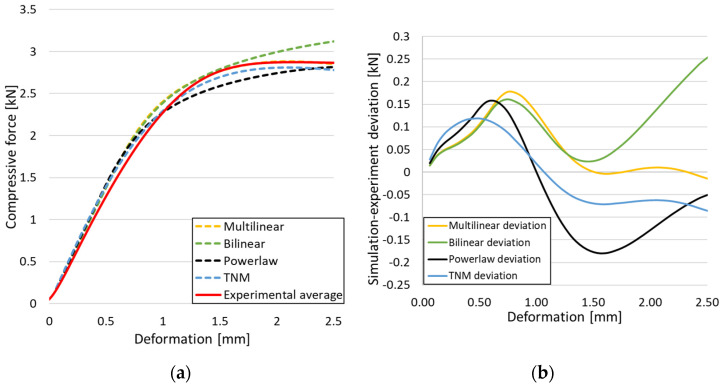
(**a**) Compressive force as a function of deformation for the proposed material models; (**b**) Difference between the dotted curves in (**a**) and the average experimental data (red curve).

Figure 9a shows the compressive force as a function of the deformation for the numerical simulations (dotted lines) obtained for the gyroid sample with the material models described in Figure 5. It is observed that all of them give a response close to the average experimental curve obtained through compressive testing of three identical samples (red curve). All models present a similar stiffness deviation in the elastic region but follow different yield responses. In order to correctly estimate which of the four models came closest to the experimental data, the difference between the area under the curve of the numerical simulations for each model and the area under the curve of the average force recorded empirically was evaluated. Figure 9b highlights this deviation. Based on this plot, deviations of 2.54% for the *Multilinear* model, 4.65% for the *Bilinear* model, 5.08% for the *Powerlaw* model, and 3.16% for the *TNM* were determined.

Thus, the *Multilinear* model presented the highest degree of curve overlap, most accurately capturing the real behavior of the samples, and was further used in the FEA.

### 3.3. Influence of Using CAD-Type Geometry or Geometry Obtained by Implicit Modeling

Considering that most of the built samples cannot be reproduced by conventional CAD means, the problem that arises is whether the use of the geometry obtained through implicit modeling introduces any errors in the FEA. For this purpose, a comparison was performed, based on the gyroid sample as a benchmark test, between the results obtained when the solid used in the numerical simulations was a conventional CAD geometry or was obtained as indicated in Figure 2. The test conditions are the same as previously stated, the only variable being the geometry type.

**Figure 10:**
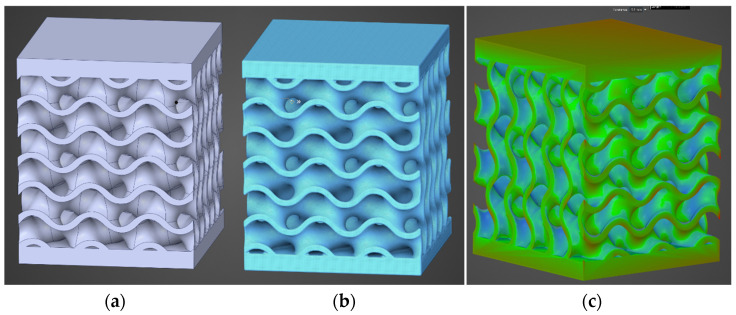
(**a**) CAD geometry; (**b**) Solid obtained from implicit modeling; (**c**) Dimensional deviations between the two formats.

Figure 10 visually shows the two formats used, the CAD one in (a), defined by the procedure presented in Figure 1, and the one obtained after correcting the geometry obtained through implicit modeling in (b), following the procedure described in Figure 2. Figure 10c displays the geometric deviations between the two models, with an imposed tolerance of 0.1 mm. This provides an overview on the fidelity of reproducing the nominal geometry through implicit modeling in order to see how these deviations may generate different outcomes in the numerical simulation.

**Figure 11:**
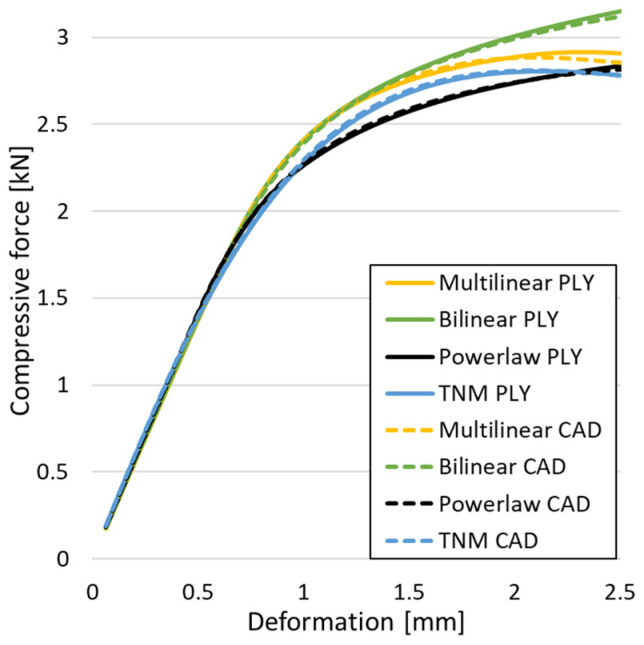
Compressive force as a function of deformation for CAD-type geometries or obtained through implicit modeling.

Figure 11 displays the differences obtained by using the two types of geometries in the computational model. For validation, all the material models presented above were verified. A very good overlap between the two pairs of tests is observed. The conclusion is that utilizing a mesh of 0.3 mm to convert the implicit model into a solid compatible with numerical simulation yields results that closely match those obtained through conventional analysis using CAD geometries.

### 3.4. Finite Element Analysis in Ansys Static Structural Module

**Figure 12:**
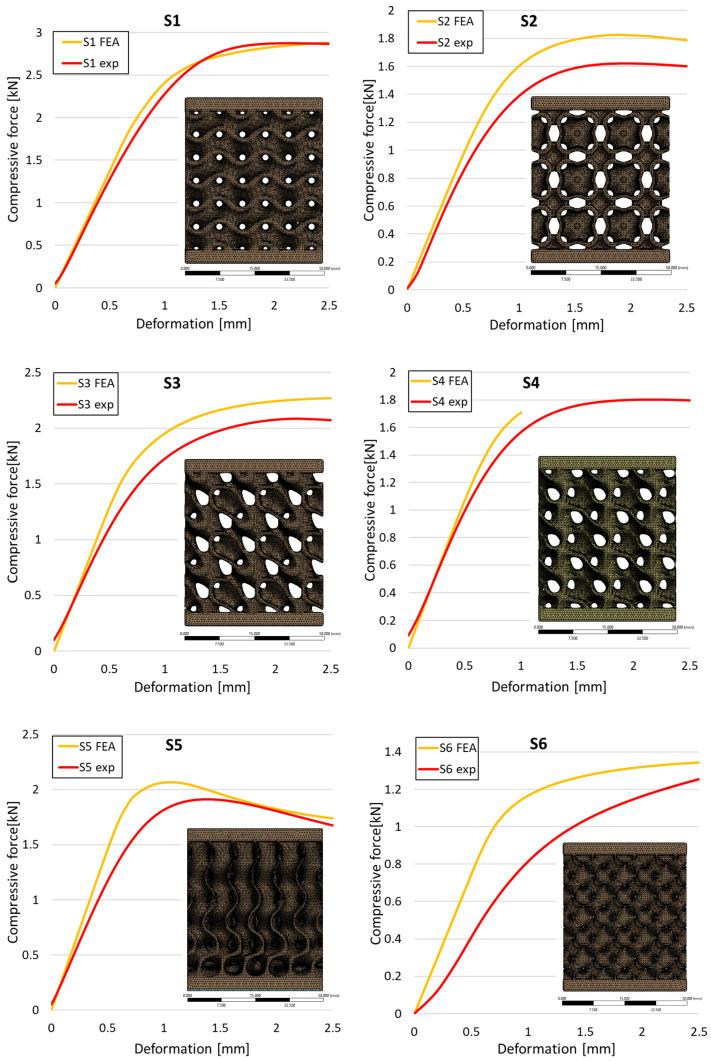

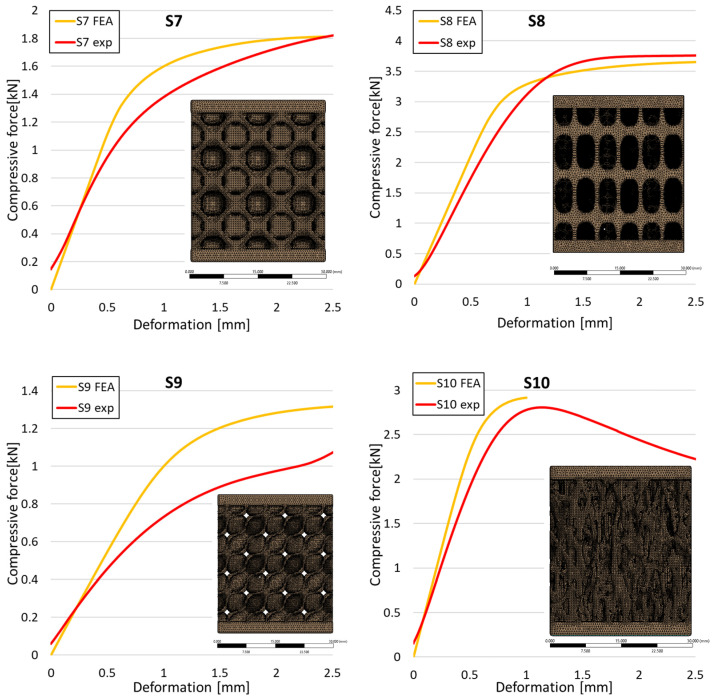
Comparison between the force–deformation curves obtained experimentally and by finite element analysis for each geometry.

The analysis of the results obtained by numerical simulation in the *Ansys Static Structural* module is presented below. Figure 12 presents the meshed geometric model and a comparison between the average force–deformation curve obtained from three experimental tests performed on each proposed structure and the curves obtained from FEA. An approach similar to that presented in Figure 9b was used to identify the degree of overlap of the curves, obtaining the following relative deviations from the empirical data: S1—1.54%, S2—13.78%, S3—10.61%, S4—7.61%, S5—8.56%, S6—29.11%, S7—8.5%, S8—1.8%, S9—30.3%, S10—10.53%. It can be seen that all samples exhibited higher FEA stiffnesses in the elastic region, which can be attributed to differences between the longitudinal modulus of elasticity obtained from the tensile tests and its simulated value. A tendency of higher simulation error from the experimental results is observed as the yield strength of the samples decreases. If geometries S1 and S8 present the highest compressive forces and the lowest deviations, at the opposite side are samples S6 and S9 with the lowest accuracies and the lowest capable forces. The high discrepancy indicated in the case of sample S6 can be attributed to the small features of the topology and the smaller wall thickness. This did not allow for three elements to be generated across the walls of the entire cell, thus reducing the accuracy of the results. In the case of specimen S9, the reason behind the high error could be its deformation mode. Due to its inclined layers, the structure does not show a bending-dominated behavior, such as for the other samples. An explicit analysis of this specimen could provide better results. At the same time, the discrepancies could also be related to alterations generated during the AM printing.

The equivalent von Mises stress criterion is specific to ductile materials such as metals. Polymers, especially under compressive loads, can exhibit complex behaviors such as viscoelasticity, non-linearity and time-dependent deformation, introducing uncertainties in the application of the criterion [48]. However, in many cases of testing parts made from plastic polymers by AM, especially for quasi-static loading, von Mises stresses are intensively used and can serve as an approximate criterion for identifying the yield phenomenon [11,27,31]. This is particularly important, especially for complex structures, where the stress states can vary significantly due to geometric complexity and three-dimensional stress distributions. Table 2 presents the distribution of equivalent von Mises stresses and equivalent total strain for the static simulations performed, along with a representation of the experimental compressive response of each structure. The yellow contour in the last column highlights the areas where visible deformation was first observed during experimental testing. The red curve indicates the failure contour of the specimen.

The distribution of equivalent stresses, in the case of specimen S1, showed high uniformity (column 2, row 1 notated as C2-R1) and together with the equivalent total strain (C3-R1), localized especially in the central area of the sample, leads to a deformation mechanism according to the one indicated in the figure in C4-R1.

Similarly, specimen S2 exhibited maximum stress values in the central layer of three-element cells (C2-R2), leading to maximum deformation in this area. This behavior is visible in the equivalent strain field (C3-R2) and the failure mode captured experimentally in C4-R2. For the S3 topology, the homogeneous stress distribution throughout the volume (C2-R3) indicates a stable failure behavior, as is visible in C4-R3. This, however, is not as easy to anticipate by analyzing only the equivalent strain field in C3-R3. Next, S4 faced convergence difficulties due to the thin walls generated on its sides and the inability to mesh them with a sufficient number of elements across their thickness. To address this, the deformation was limited to 1 mm, which only captures the elastic behavior of the specimen and the onset of yielding. Even at low deformation values, the regions with maximum equivalent stresses (C2-R4) coincide with the areas where deformation was first observed during experimental testing (C4-R4). Similarly, the equivalent total strain in C3-R4 indicates the critical regions between the layers, where yielding led to layer self-contacts, as highlighted in C4-R4. For specimen S5, the failure mechanism is easier to anticipate, with maximum equivalent stresses concentrated in the upper region (C2-R5) consistent with the deformation mode presented in C3-R5. Specimen S6 demonstrated the best uniformity, both in terms of equivalent von Mises stresses (C2-R6) and equivalent total strain (C3-R6). In this case, it was difficult to estimate the failure mode identified in C4-R6, with higher strain values in the lower zone of the specimen. For S7 the figures in C2-R7 show a leftward shift of the equivalent von Mises stresses, together with higher strain values in those zones suggested by C3-R7. Both these results are consistent with way the structure loses its equilibrium, identified experimentally in C3-R7, generated by buckling along the middle of the vertical walls of the RVE. As expected, specimen S8 presents the highest strains in the thin areas generated along the contour, which fractured at a specific strain of 20%. The distribution of equivalent von Mises stresses (C2-R8) and the most deformed areas (C3-R8) corresponded to empirical observations of 45° deformation angles on the outer walls (C4-R8). Topology S9 experienced the highest values of equivalent von Mises stresses (C2-R9) and equivalent total strain (C3-R9) in the areas between the inclined layers that make up the geometry, resulting in a folding failure mechanism where cells lay one on top of the other, as shown in C4-R9. Finally, specimen S10 also encountered convergence difficulties due to its stochastic nature, limiting the simulation to a 1 mm deformation. For this geometry, identifying a failure mechanism cannot be a realistic resolution, as the maximum equivalent stresses and total strains exhibited unpredictable behavior. Consequently, a direct comparison between experimental and FEA results was not feasible for this sample.

### 3.5. Finite Element Analysis in Ansys Explicit Dynamics

Next, the numerical simulation was conducted using the Ansys Explicit Dynamics module to evaluate the deformation mechanism across the entire range of experimentally imposed deformations. This type of FEA can handle cases of significant shape changes or lack of linearity of the material more efficiently.

Table 3 shows the variations in compressive force as a function of deformation, obtained experimentally and via FEA in both the *Static Structural* and *Explicit Dynamics* modules for the S1 and S8 topologies. The gyroid, S1, was selected as a reference due to its significance in relevant literature, while topology S8 was validated for demonstrating superior yield strength compared to the gyroid. Considering the constant input parameters of the two simulations, the overlap on the first 2.5 mm is evident. The slight difference between the two FEA curves (yellow and green) may be due to two factors. First, the analysis performed in the *Explicit Dynamics* module has a larger deformation increment, which can lead to less accurate approximations of the curve between successive points. Second, the definition of the *Multilinear* material is limited to a maximum of 10 points.

The proposed model does not adequately capture the non-linearity caused by the hardening phenomenon in the samples, as indicated by the reduced loading force at high strain values in both green curves. The figures in column C2 display the equivalent von Mises stress fields at a deformation of 10 mm. The general failure mechanism is similar to the one identified experimentally and presented in Table 2, column C4.

## 4. Discussion and Conclusions

After conducting a sensitivity analysis of the geometric model, it was determined that a maximum size of 0.3 mm for the regular tetrahedron elements offers an optimal balance between result accuracy and computational efforts. While this value is in line with similar sensitivity analyses from relevant literature [49,50], the analysis was specifically conducted to validate the mesh size for the proposed TPMS structures, given their intricate geometry and different dimensions. This corresponds to at least three elements along the thickness of the walls of the cellular solids. However, employing a uniform meshing strategy throughout the entire part may prove to be insufficient in the case of geometries with walls whose thickness is not constant or in areas with very low thicknesses, where at least two rows of elements cannot be generated. This, in turn, results in convergence problems or accuracy reduction, as observed in the cases of topologies S4 and S10.

An analysis was conducted on the implications of using implicit modeling in FEA. For the same gyroid topology, S1, numerical analyses were carried out using geometric models created through both conventional CAD methods and implicit modeling. The dimensional deviations between the models exceeded the imposed tolerance value of 0.1 mm, with maximum values of 0.17 mm. However, the resulting variation of compressive force as a function of deformation showed a very good overlap between the two models. A disadvantage of using geometries obtained through implicit modeling is the significantly longer time required to establish the faceted geometry, transform the geometry into a solid, perform the meshing, and solve the model. This can be up to 4 times longer than in the case of a conventional approach, which makes the finer meshing of solids more challenging due to the rapid increase in computational effort.

Four material models were proposed to identify which best approximates the behavior of the selected photopolymer resin. Data obtained from tensile tests were used to empirically define two isotropic material models: *Bilinear* and *Multilinear*. Following this, an iterative method was employed to configure two additional material models: *Powerlaw* and the *Three-Network Model*. The parameters defining their equations were configured so that the characteristic curves of the materials overlapped as accurately as possible over the average true stress–strain curve obtained through tensile tests. FEAs were conducted for all four material models, and the error between the FEA results and the experimental data determined through three different compression tests was assessed. Deviations of less than 6% were found, with the *Multilinear* model achieving the best accuracy at only 2.54% deviation. This is in line with findings in relevant literature for isotropic hardening models such as the analyses performed by Zheng et al. in [10], Naghavi et al. in [30], or Krešic et al. in [51]. Also, as suggested by these studies, the numerical results for compressive force after yielding tends to have lower values than the experimental data.

Performing quasi-static numerical analyses that include high strain values on complex three-dimensional geometries can prove difficulties due to the contact interactions within the samples. Increasing the tolerance of the walls penetrating each other can increase the convergence rate but will also lead to reduced accuracy. In this sense, static tests were performed for a deformation of only 2.5 mm, a value chosen so that there would be no contacts that would lead to convergence difficulties. Even in this case, due to the periodic nature that determined thin walls on specimen S4 and the stochastic nature of sample S10, the deformation value was reduced to 1 mm to achieve convergence, even though this limited the range of phenomena captured.

By corroborating the equivalent von Mises stresses and equivalent total strain fields obtained for each sample, conclusions were drawn regarding the general failure modes of the samples. In most instances, the FEA results indicated a deformation mechanism similar to what was observed in experimental testing. However, sample S6 was an exception, as the substantial deformation occurring in the lower part of this specimen was not captured in the numerical simulation. This discrepancy could be due to the limited strain value imposed in the simulation, or because the real failure mode was influenced by manufacturing defects which were not considered in the FEA.

Using implicit models in FEA is a viable approach when traditional CAD models are unavailable due to complex designs. The numerical simulation method proposed in this paper automatically generates or optimizes innovative topologies through implicit modeling, allowing for the validation of their properties before manufacturing takes place. This process will be the focus of a future study, which will involve the automatic generation and evaluation of structures using a Python–Ansys interface.

## Figures and Tables

**Table 1:** Parameters used to define the material models used in FEA.

**Bilinear isotropic hardening model**		**Multilinear isotropic hardening**	
Young‘s modulus *E* = 1296 MPa		$\varepsilon_{el}=\frac{\sigma_{real}}{E}$	(1)
Yield strength $\sigma_{y}$ = 32.12 MPa		$\varepsilon_{pl}=\varepsilon_{real}-\varepsilon_{el}$	(2)
Tangent modulus *E*_t_ = 90.12 MPa		$\sigma_{real}=138{\varepsilon_{pl}}^{2}-6.7\varepsilon_{pl}+35$	(3)
**Power law non-linear isotropic hardening**		**Three-network model [24]**	
$\frac{\sigma_{real}}{\sigma_{y}}={\left(\frac{\sigma_{real}}{\sigma_{y}}+\frac{3G}{\sigma_{y}}\varepsilon_{pl}\right)}^{N}$	(4)	Shear modulus A—140.5 MPa	Shear modulus evolution rate B—16
		Flow resistance A—51.5 MPa	Flow resistance B—76.5 MPa
*N* = 0.15 [41]		Stress eponential A—1.4	Stress eponential B—2.1
		Initial shear modulus B—362.5 MPa	Shear modulus C—2.5 MPa
		Final shear modulus B—22.5 MPa	Locking stretch—16 Bulk modulus—502 MPa

**Table 2:** Visualization of FEA results and experimental compressive response for each sample.

Sample (C1)	Von Mises Equivalent Stress Distribution [MPa] (C2)	Total Strain Distribution [-] (C3)	Compressive Response (C4) [34]
S1 (R1)	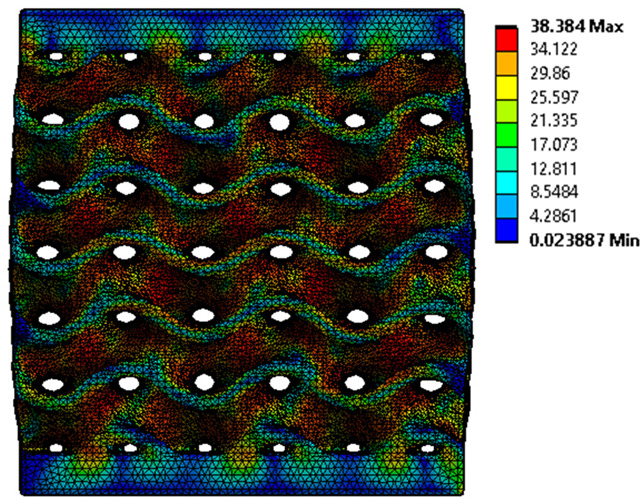	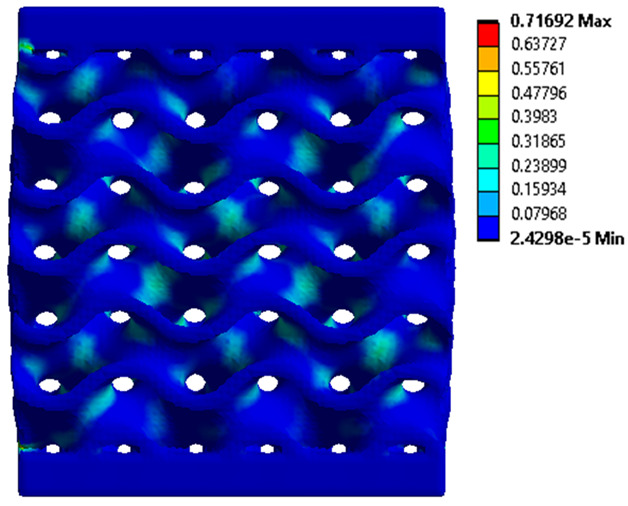	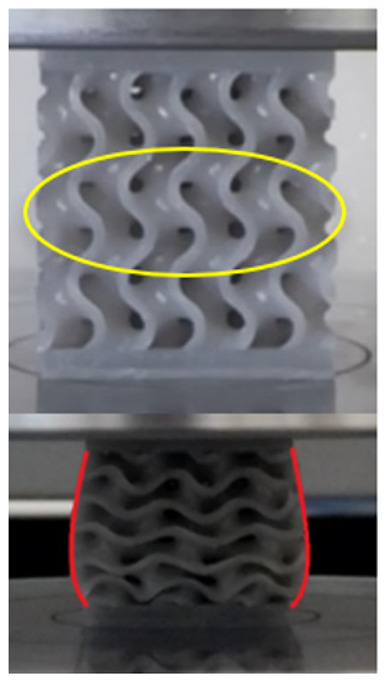
	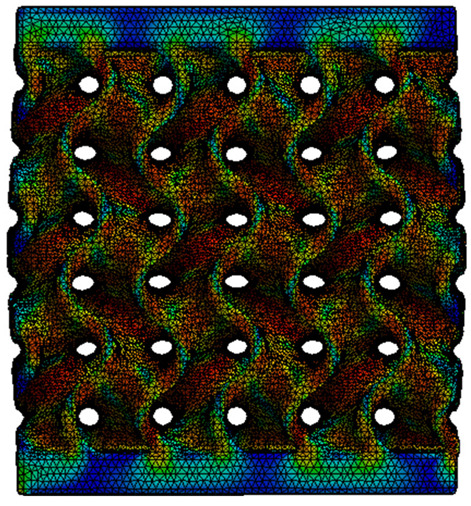	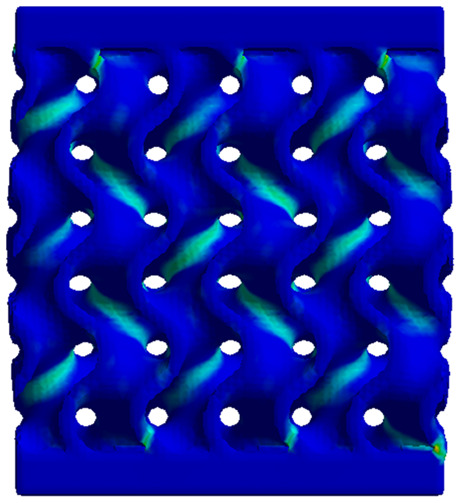	
S2 (R2)	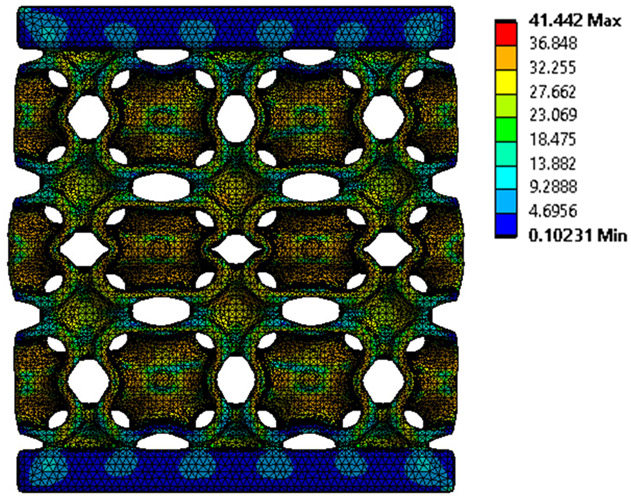	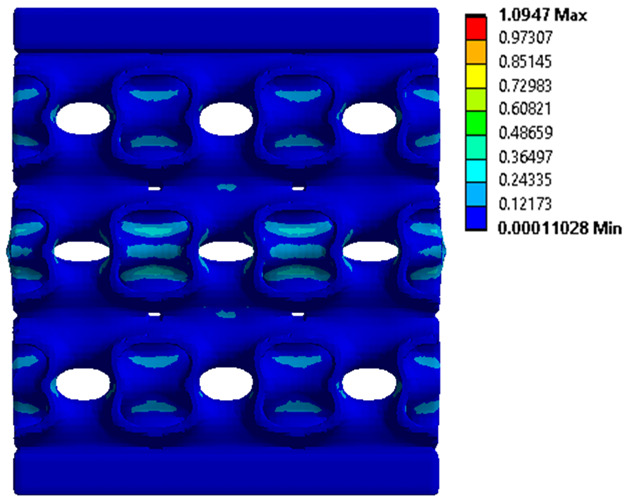	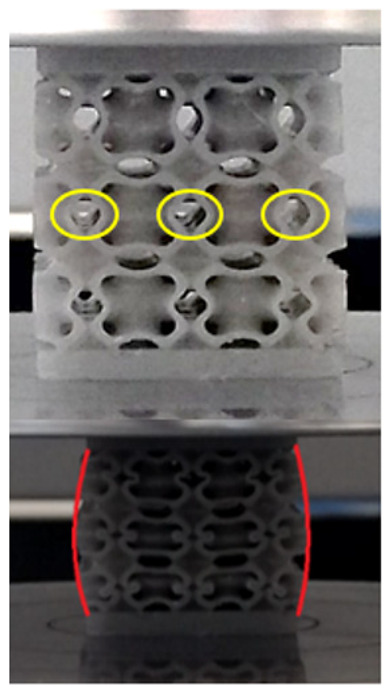
	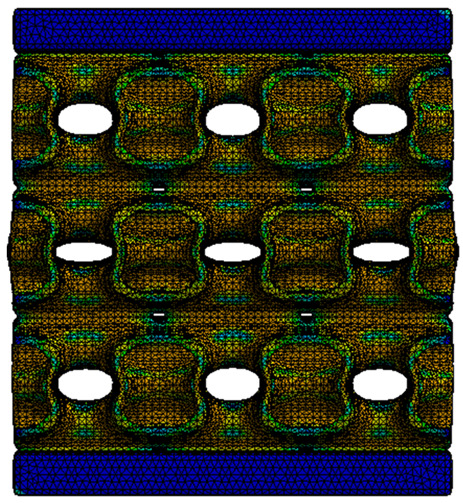	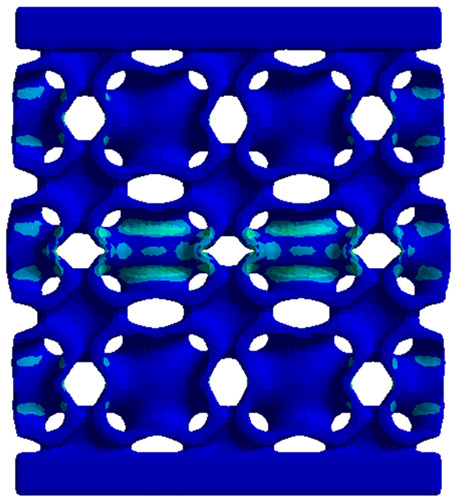	
S3 (R3)	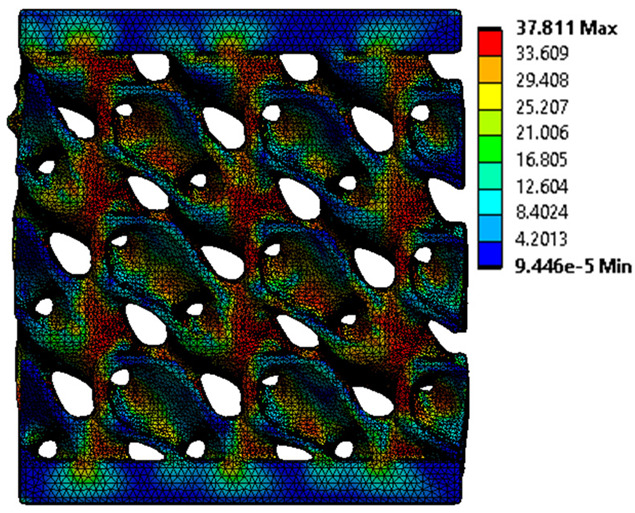	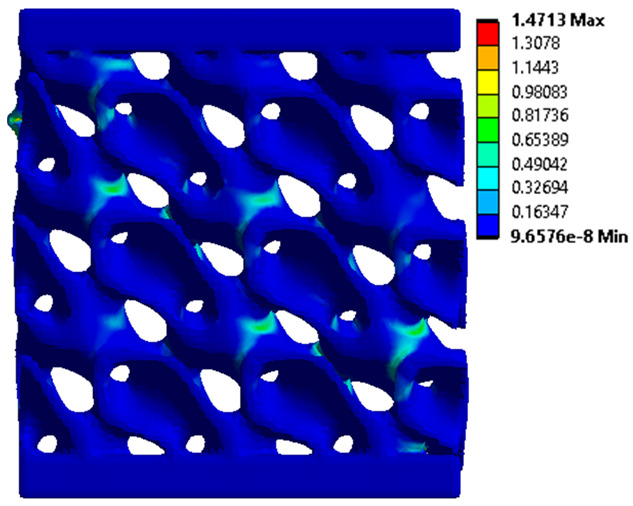	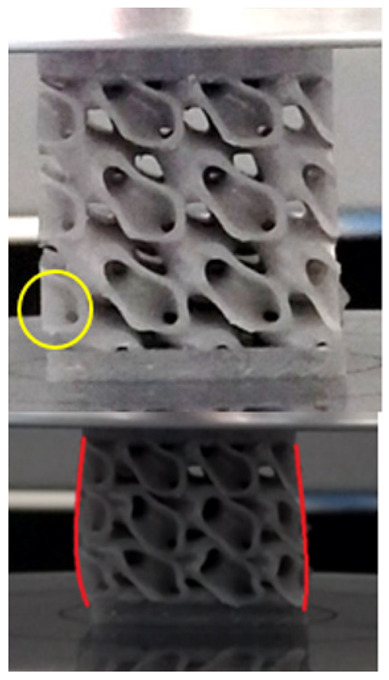
	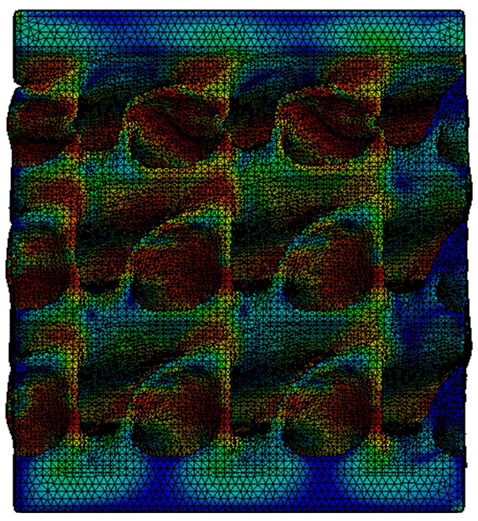	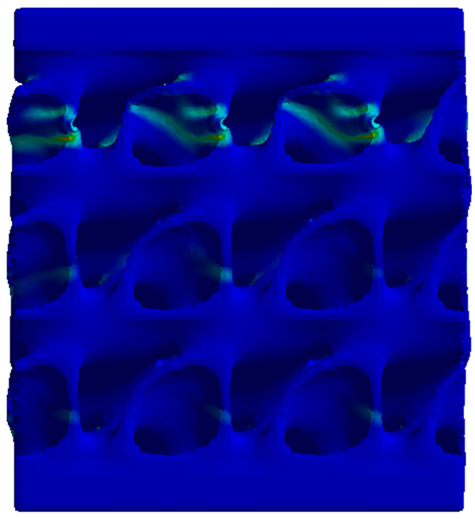	
S4 (R4)	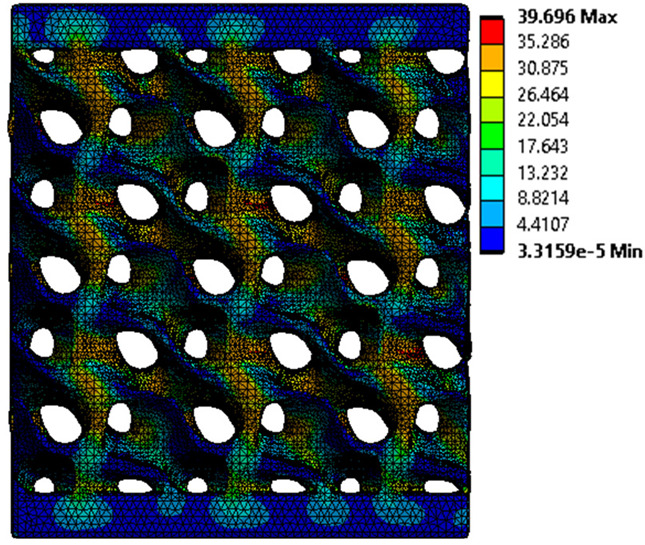	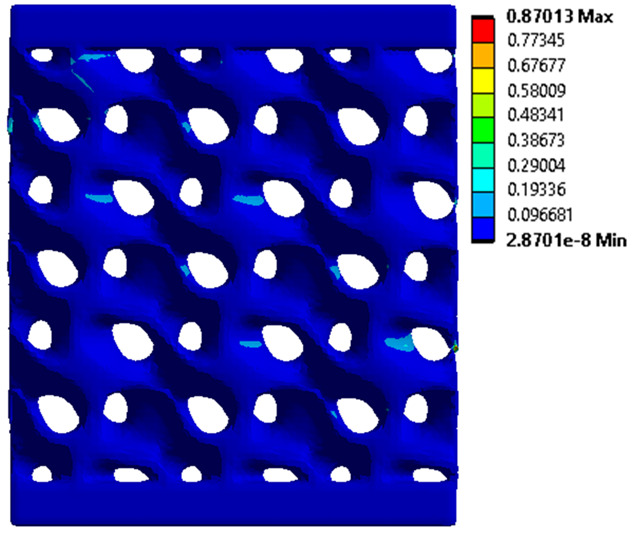	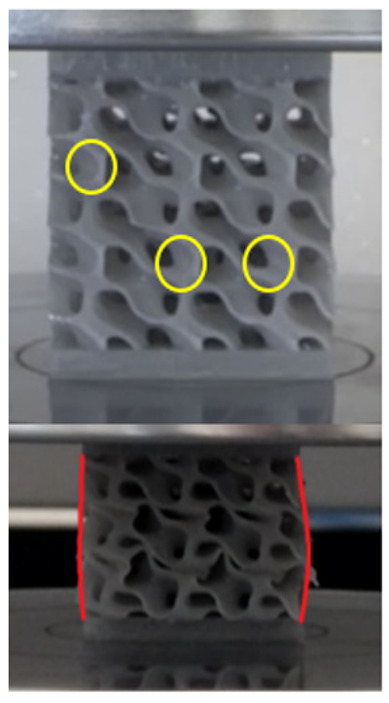
	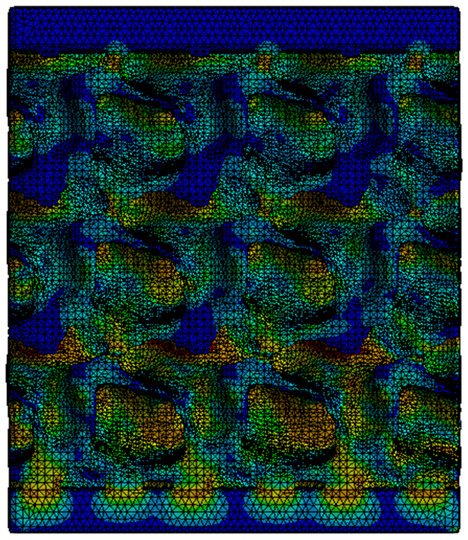	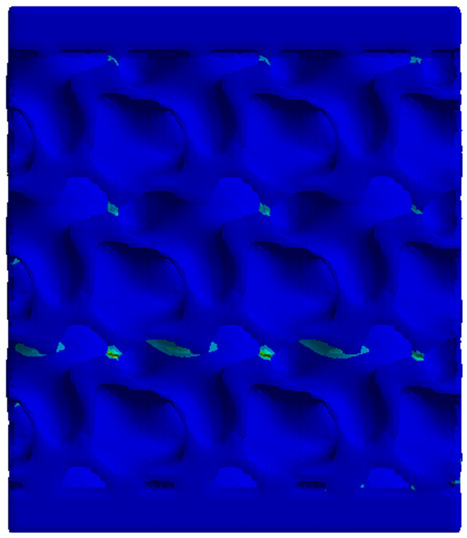	
S5 (R5)	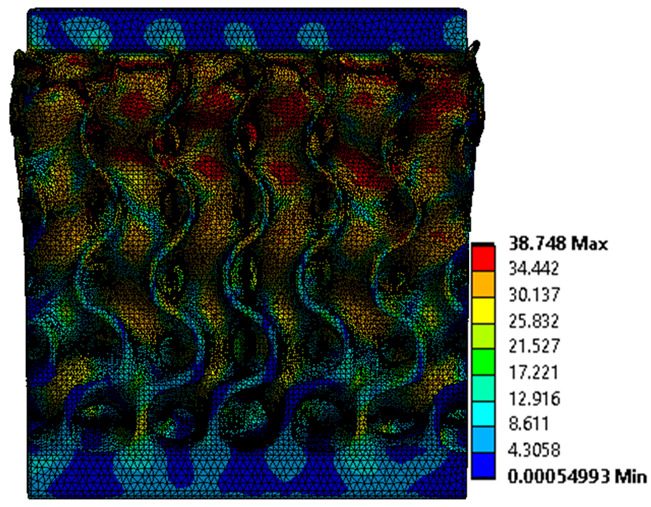	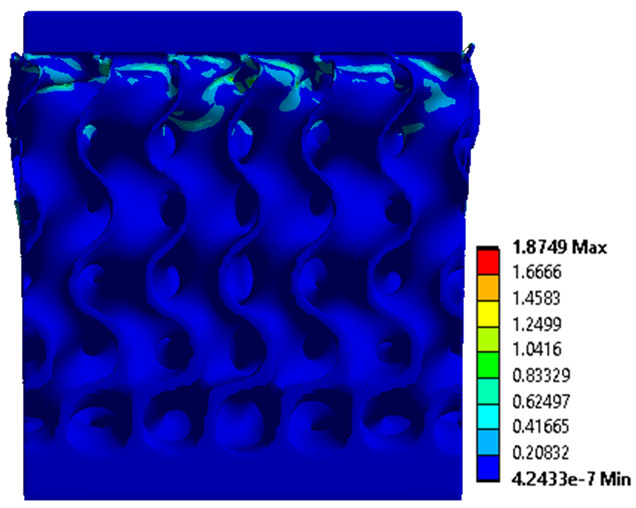	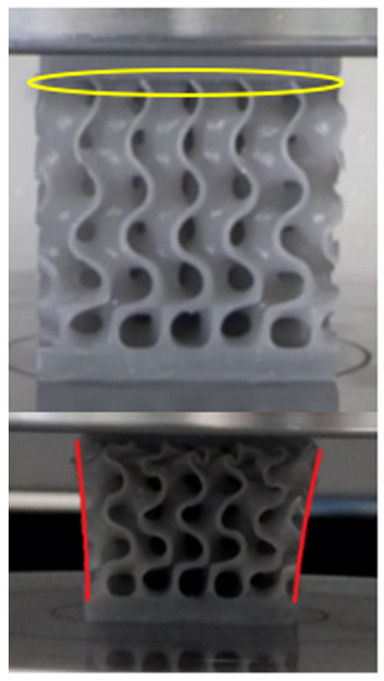
	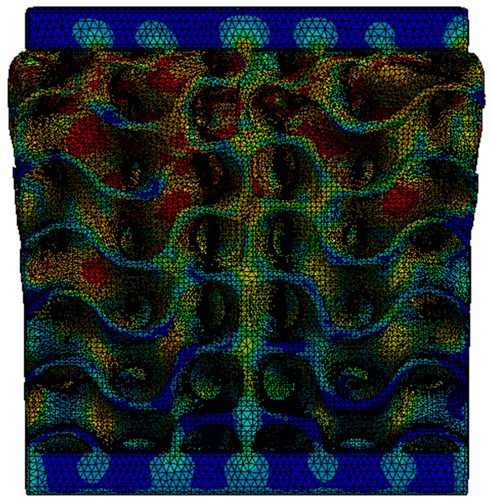	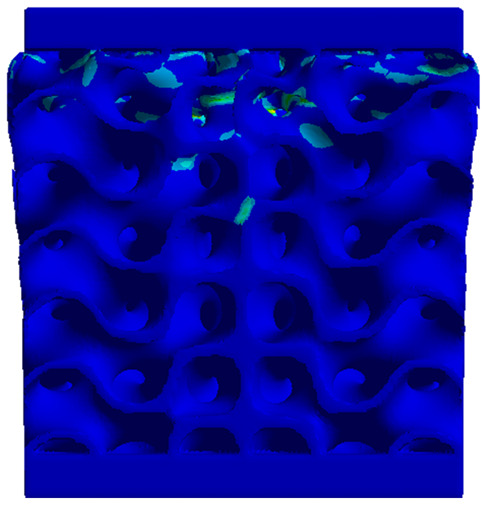	
S6 (R6)	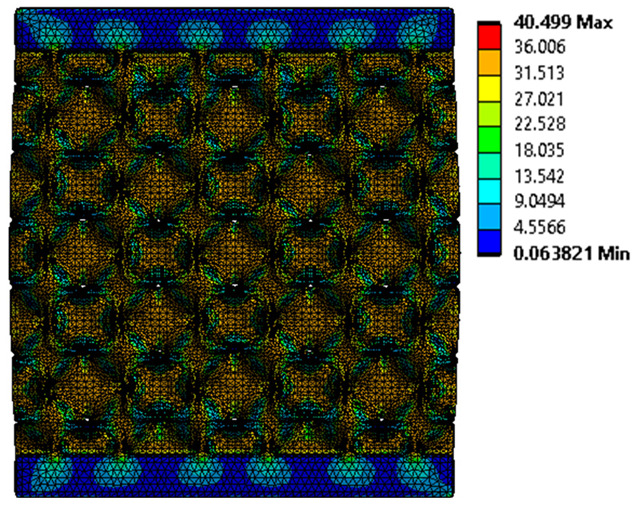	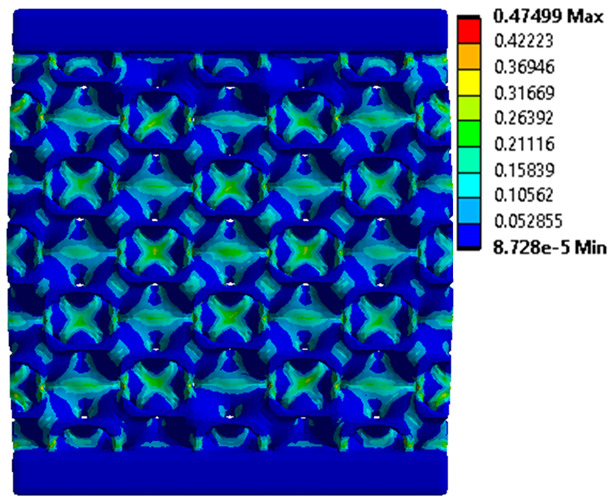	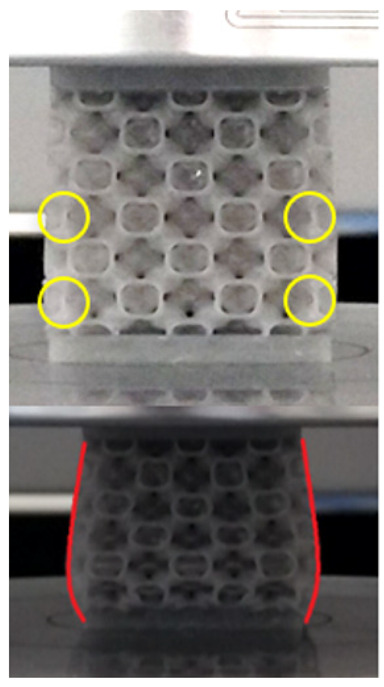
	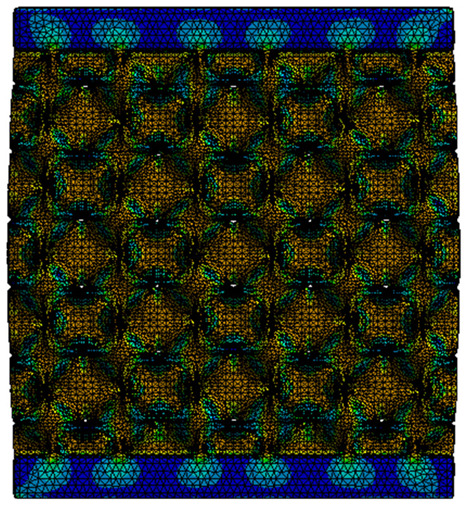	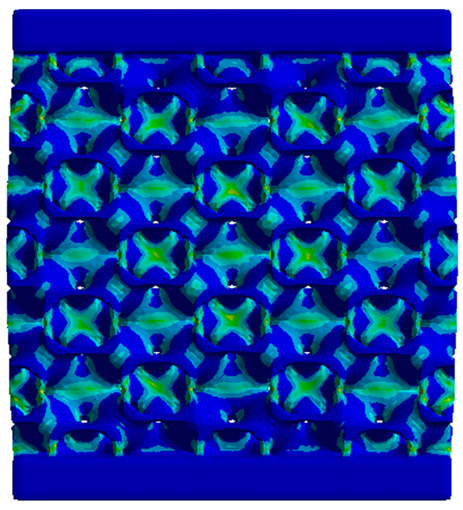	
S7 (R7)	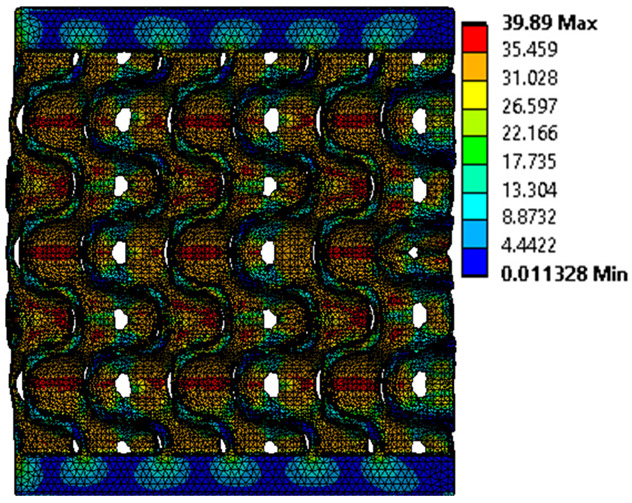	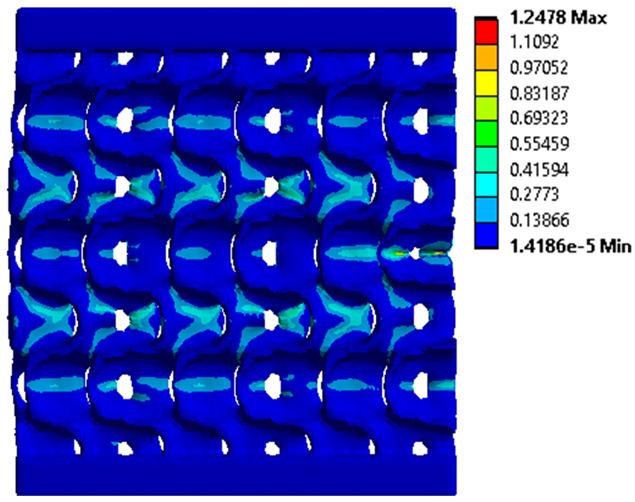	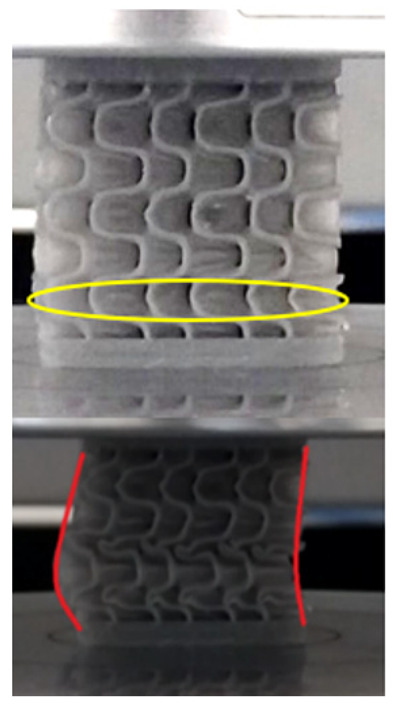
	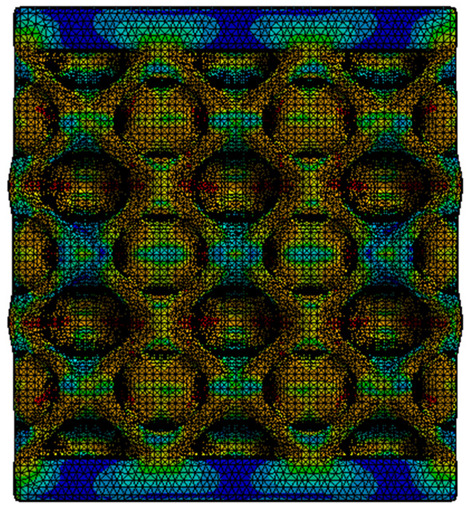	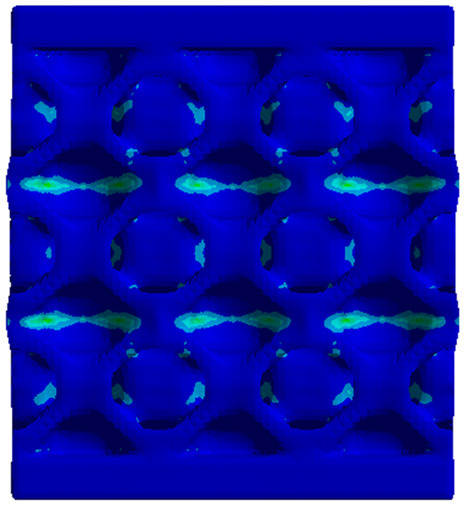	
S8 (R8)	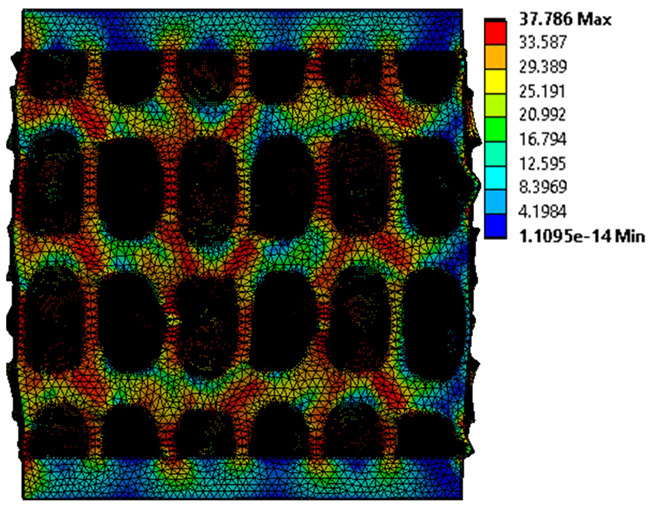	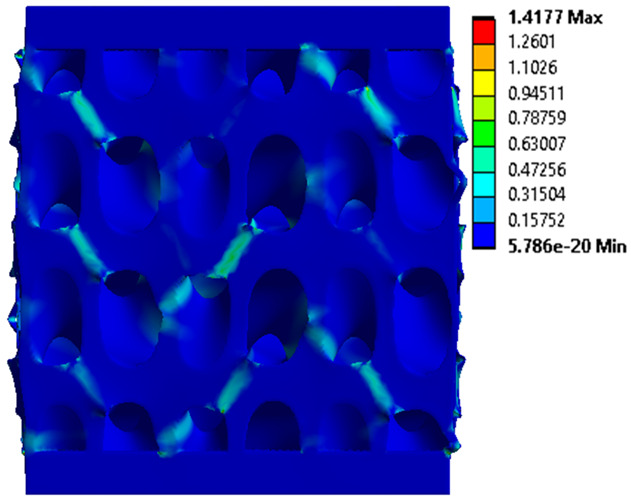	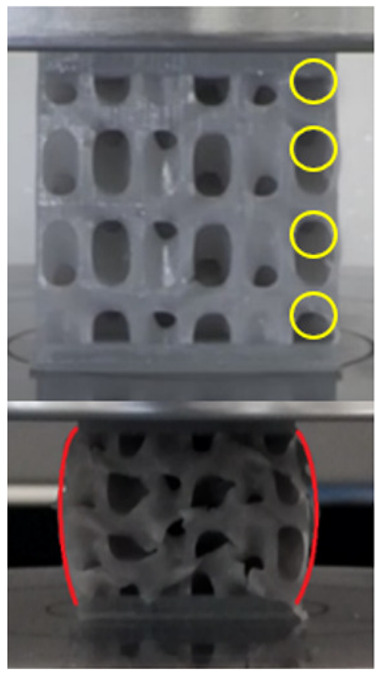
	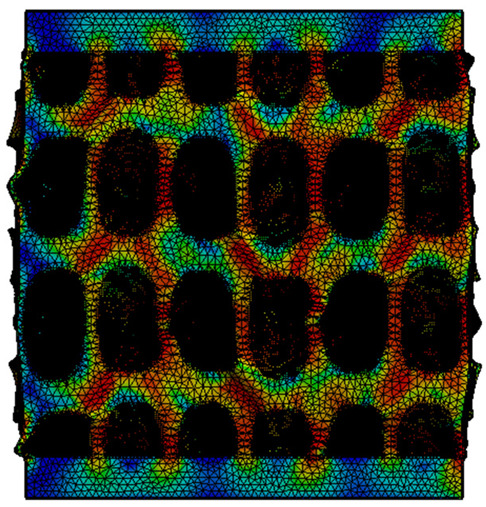	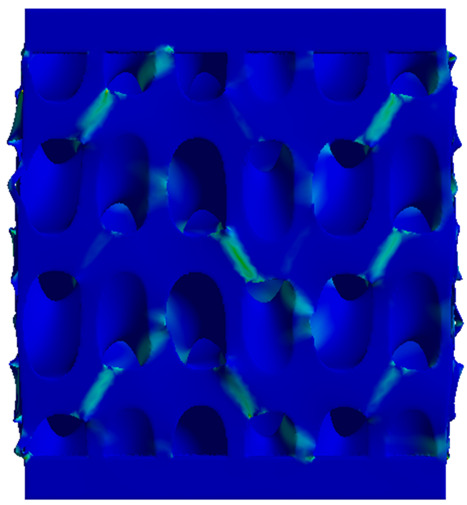	
S9 (R9)	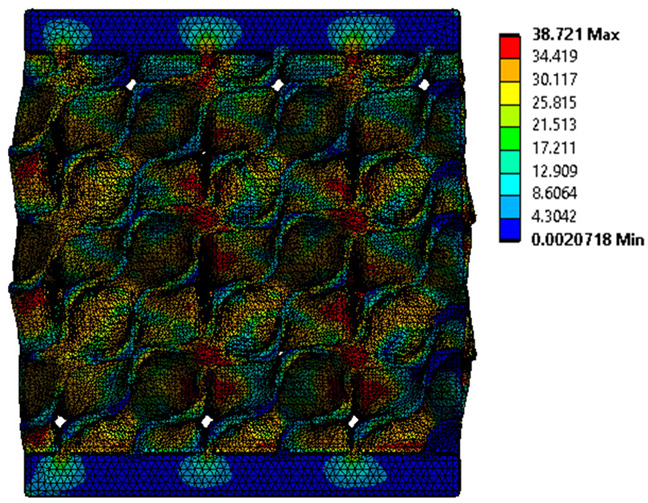	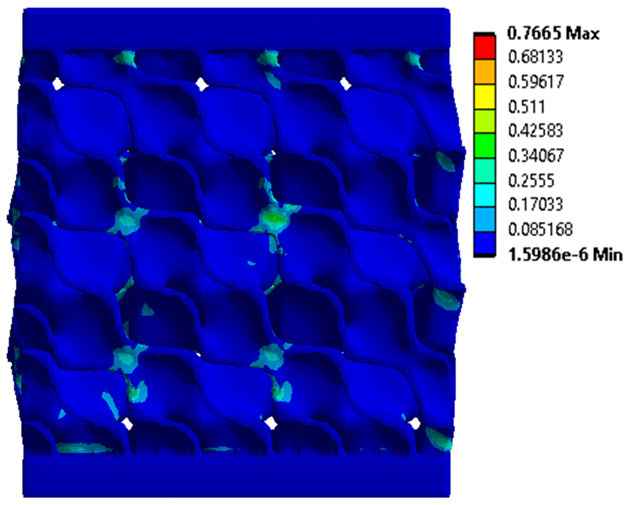	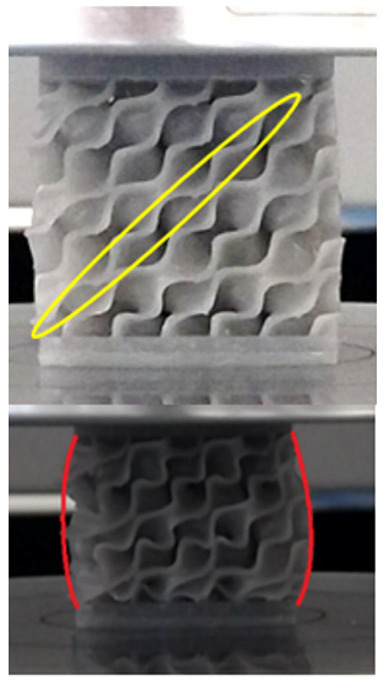
	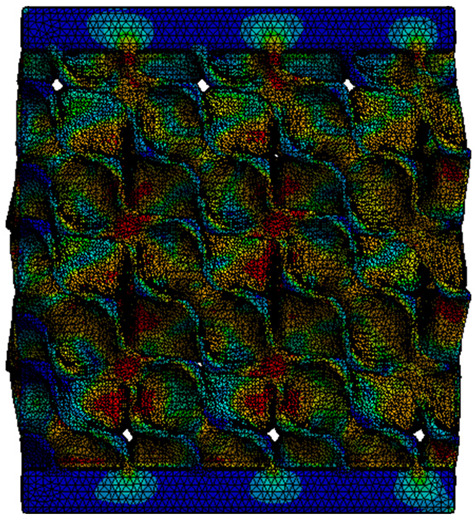	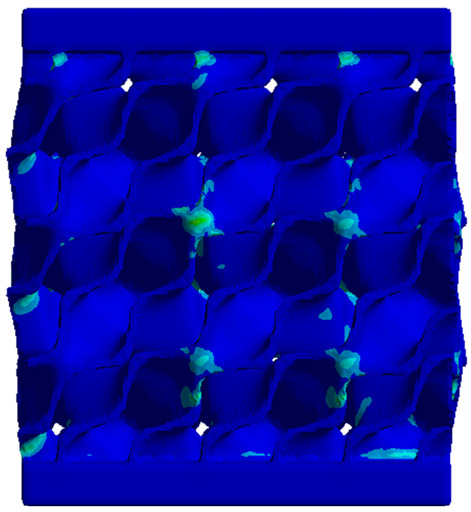	
S10 (R10)	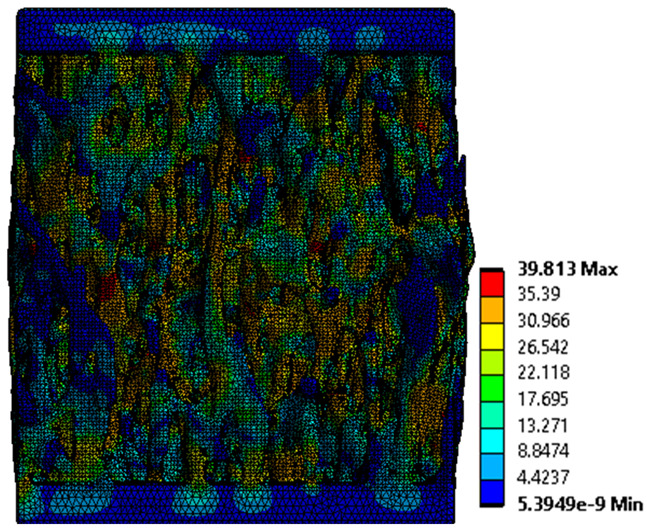	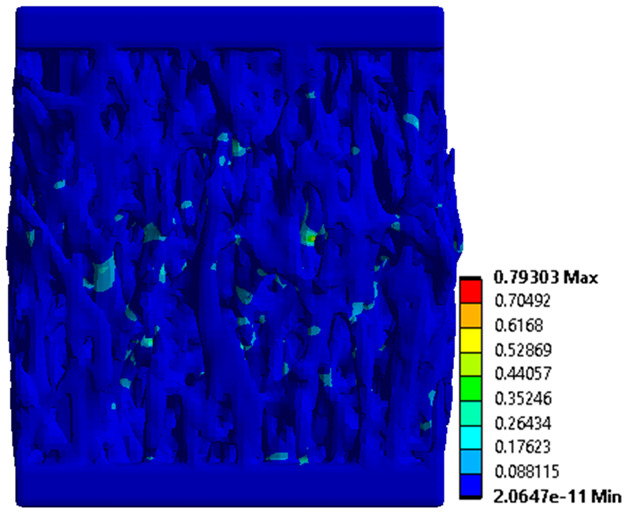	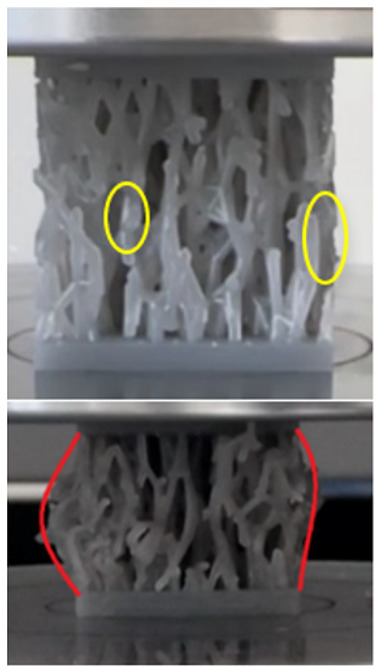
	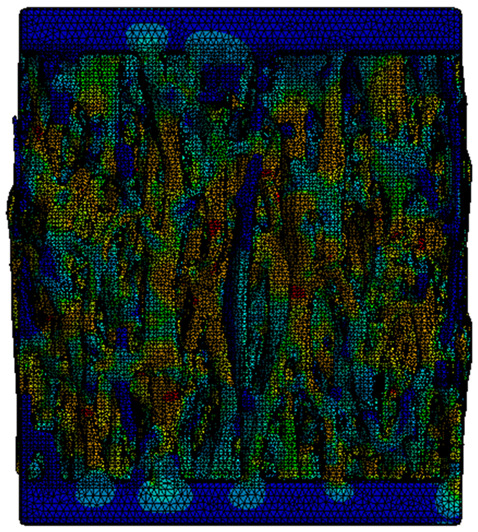	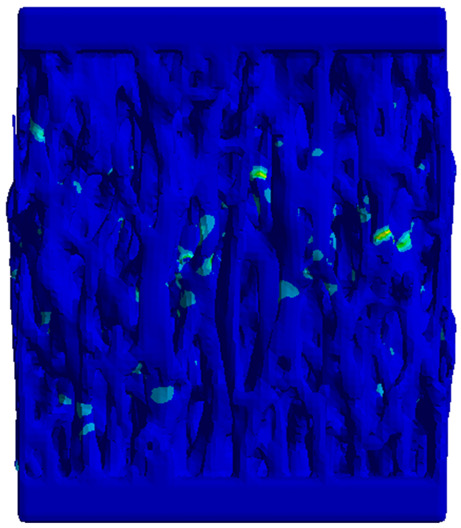	

**Table 3:** Visualization of FEA results and experimental compressive response for samples S1 and S8.

Sample (C1)	Von Mises Equivalent Stress Distribution [MPa] (C2)	Comparison Between the Force–Deformation Plots and Those Obtained by Finite Element Analysis (C3)
**S1** **(R1)**	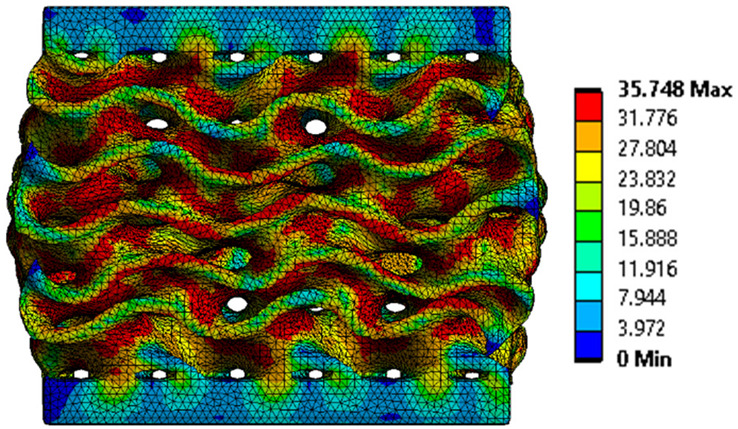	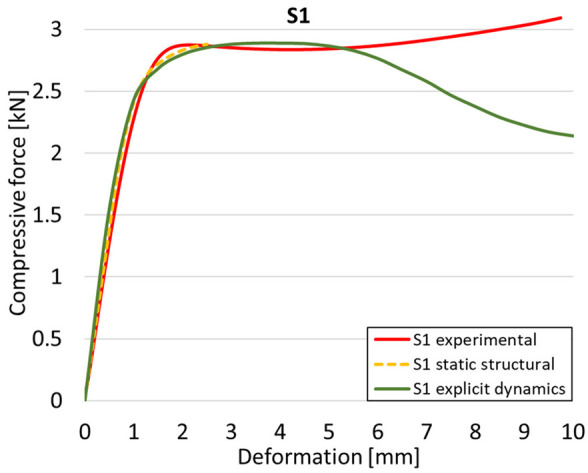
	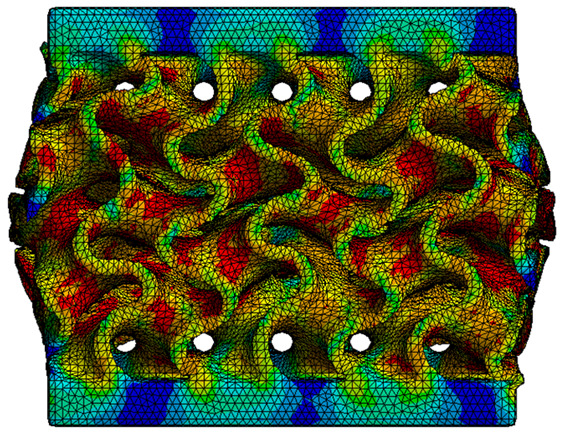	
**S8** **(R2)**	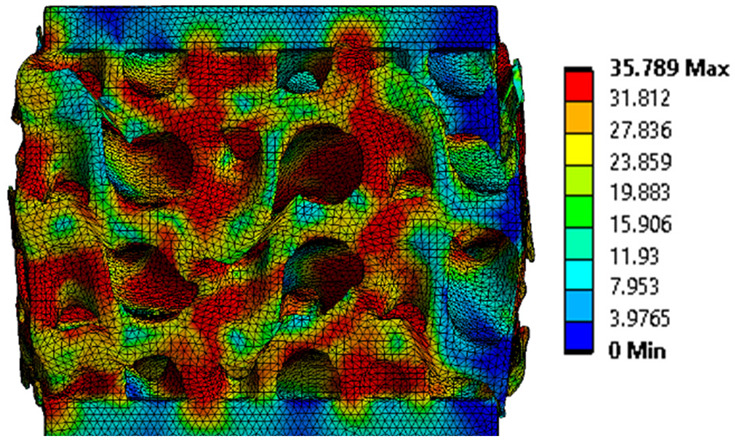	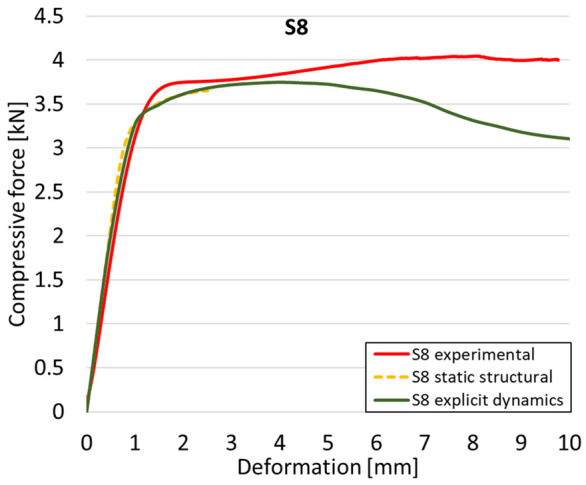
	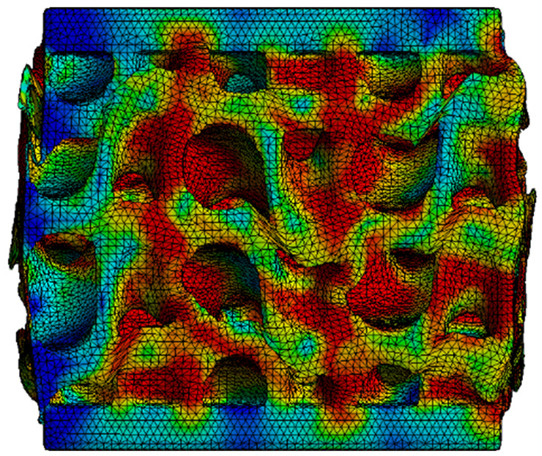	

## Data Availability

The original contributions presented in the study are included in the article, further inquiries can be directed to the corresponding author.
